# Longitudinal and Concurrent Changes in Brain and Gut due to Morphine Self‐Administration

**DOI:** 10.1111/adb.70059

**Published:** 2025-06-10

**Authors:** Kaylee Brunetti, Zicong Zhou, Samia Shuchi, Raymond Berry, Sabrina White, Yan Zhang, Michael S. Allen, Shaohua Yang, Johnny D. Figueroa, Luis Colon‐Perez

**Affiliations:** ^1^ Department of Pharmacology and Neuroscience University of North Texas Health Science Center Fort Worth Texas USA; ^2^ Department of Microbiology, Immunology & Genetics University of North Texas Health Science Center Fort Worth Texas USA; ^3^ Center for Health Disparities and Molecular Medicine and Department of Basic Sciences, Physiology Division, Department of Basic Sciences Loma Linda University Health School of Medicine Loma Linda California USA

**Keywords:** addiction, diffusion MRI, gut, IV self‐administration, microbiome, morphine, small animal imaging, striatum

## Abstract

Opioid agonists are known for their effects on the opioid and dopaminergic systems; however, new research points to complementary changes in the gut underlying maladaptive changes associated with opioid use. The gut–brain axis (GBA) is a bidirectional signaling process that permits feedback between the brain and gut and is altered in subjects with opioid use disorders, but the spatiotemporal correspondence between quantitative translational measures of gut and brain health is not clear. In this work, we determined longitudinal and concurrent changes in the brain and gut of rodents trained to self‐administer morphine for 14 days. Active lever presses delivered a single infusion of morphine (0.4 mg/kg/infusion). We used MRI and 16s rDNA analysis of faecal matter to identify changes from baseline (naïve, nondrug state) to an acute phase (early in the self‐administration process, after 2 days of self‐administration) and a chronic phase (late in the self‐administration process, after 14 days of self‐administration). Animals were scanned in a 7T MRI scanner three times (baseline, acute and chronic), and before scanning, faecal matter was collected from each rat. We found early changes in gut microbiota diversity and specific abundance as early as the acute phase that persisted into the chronic phase. In MRI, we identified alterations in diffusivity indices both within subjects and between groups, showing a main effect in the striatum and thalamus. We posit that gut changes precede the effects observed in MRI, with the striatum and thalamus emerging as crucial links mediating communication between the gut and the brain.

## Graphical abstract

With this work, we present evidence of longitudinal changes in the brain and gut microbiome following morphine use. We used a within‐subjects design to show that chronic administration of morphine alters 1) the striatum microstructure using MRI, 2) gut microbiome diversity derived from fecal samples, and 3) these changes are correlated to each other. Our findings further show evidence of gut‐brain bi‐directional communication, and this signal may be an important biological mechanism mediating the use and misuse of opioids.

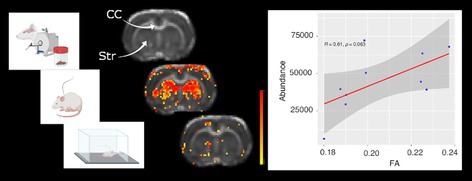

## 1 Introduction

Opioid use disorder (OUD) is a health care problem in need of novel approaches for its effective treatment [1, 2]. A potential new approach is to concomitantly target the peripheral and central nervous system (CNS) effects of opioid agonists. On the one hand, opioid agonists act on the CNS more specifically in the brain in neurons expressing opioid receptors. The brain alterations due to opioid use are often in the striatum [3], thalamus [4] and among other brain regions of the brain reward circuitry of great significance to the psychopathology of OUD [5, 6]. On the other hand, opioid receptors are also widely expressed in the gastrointestinal tract, affecting gastrointestinal function. Gastrointestinal opioid receptor activation stimulates smooth muscles to reduce motility, leading to opioid‐induced constipation and disruptions of the tight junctions in the gut leading to infiltration of pathogens in the body [7, 8, 9]. Moreover, peptides associated with appetite regulation are expressed throughout the brain's reward circuitry [10], implying a synergy in the neurobiological mechanisms between food and opioid consumption [11]. The synergy between the gut and brain has been described as a bidirectional communication between the brain and the gut [12, 13]. Recently, there has been an interest in studying OUD through a gastrointestinal and brain disturbances view, known as the gut–brain axis (GBA); however, critical spatiotemporal alterations in the gut and brain associated with the transition from a naïve to an opioid user are not well elucidated.

Morphine's simultaneous effects in the periphery and the CNS make it necessary to determine the impact one organ has on the other to identify concomitant pathological trajectories affecting the brain and gut. Magnetic resonance imaging (MRI) allows us to probe the neurobiological changes of opioid use and the temporal features associated with morphine use [14]. Contrastingly, new sequencing technologies, like 16S rDNA sequencing of faecal matter, allow us to study the gut and its microbiome in vivo and noninvasively, yielding instrumental insights to understand the relationship between opioid agonists use and the gut [15]. Crucial to gaining insights of temporal trajectories associated with extended use of opioids and morphine is being able to track individuals as they consume morphine or opioid agonists. MRI, 16S rDNA, along with volitional models of morphine use, such as self‐administration, allows us to gain new insights regarding the spatiotemporal nature of brain changes due to morphine use. Self‐administration represents the gold standard in substance use disorders, often used in animal studies of drug use but not in the gut microbiome. Self‐administration allows us to probe chronic (i.e., more than daily 6 h and 4 weeks of drug access, between known as extended access) to subchronic effects of morphine (i.e., between 1 and 3 daily hours and between 1 to 2 weeks of access to drugs, better known as short access) on the brain and the gut. Both drug use paradigms represent distinct aspects of detrimental morphine use, such as risky recreational (subchronic) and pathological (chronic). Combining neuroimaging and 16S rDNA sequencing is a critical need to determine the concurrent neurobiological alterations due to opioid use that can be related to simultaneous changes in gut composition associated with the use of opioid agonists; however, neuroimaging and gut diversity have *not* been explored in the context of morphine use.

Given the ongoing evolution of gut health studies and the role of microbiome diversity in substance use disorders, it is crucial to assess the trajectories of concurrent effects of opioid agonists on the gut and brain. This article utilizes animals' self‐administering morphine subchronically to determine concurrent and longitudinal alterations in gut microbiome and brain features measured with dMRI and 16S rDNA. The striatum and thalamus display a correlation to changes in the gut. This work presents links for the hypothesis that the brain and gut continuously communicate with one another when consuming morphine. Further expanding our knowledge on GBA by utilizing neuroimaging in animal models of volitional substance use could provide new insights into the simultaneous changes in the gut and brain due to morphine use that could serve as new models to inquire about new interventions aimed at alleviating the effects of opioid use.

## 2 Methods

### 2.1 Animals

Adult male (*n* = 13) and female (*n* = 13) Sprague Dawley rats were purchased from Charles River Laboratories (Raleigh, North Carolina). The Department of Laboratory Animal Medicine at the University of North Texas Health and Science Center provided care for the rats in this study, and the Institutional Animal Care and Use Committee at the University of North Texas Health and Science Center approved all experiments (#: 2022‐0010). Experimenters were blinded for the MRI analysis and faecal processing and unblinded for the final statistical analysis. Animals were euthanized within 2 days of the last MRI session, and brains were rapidly removed, fixed in 4% formalin and further processed to be embedded in paraffin.

### 2.2 Surgery and Catheter Implantation

Rats were quarantined for roughly a week before undergoing catheterization surgery. Rats were anaesthetized with isoflurane (5% induction, 2% maintenance) and administered subcutaneously Meloxicam (1 mg/kg). The right jugular vein was ligated with a catheter and secured in place using aseptic surgical techniques [16]. The jugular catheters were constructed in‐house with MRI‐compatible cannulas (P1 Technologies, Roanoke, VA). Rats were given 5 days to recover from surgery, after which they were shaped for morphine or sucrose pellet self‐administration. Catheters were checked for patency by observing a rapid and transient loss of muscle tone following an infusion of 0.1 mL of propofol.

### 2.3 Intravenous Self‐Administration of Morphine

Self‐administration operant behaviour was performed in eight operant chambers (MedAssociates, Fairfax, VT, USA). The chambers were equipped with two retractable levers and a variable speed syringe pump for infusions. The Drug Supply Program of the National Institute on Drug Abuse supplied the morphine used in this study. Morphine hydrochloride was dissolved in 0.9% sterile saline (0.4 mg/kg/infusion). Animals were randomly assigned to either group prior to the start of the experiment. Animals were pretrained for two 2 days to learn that lever pressing results in a reward of a single sugar pellet for 30 min and alternating lever on each day. On the third day, animals went on to self‐administer morphine under an FR‐1 schedule for 2 h daily for 14 days (weekdays on, except when rats were due in the MRI, and weekends off). During self‐administration sessions, only one of the two levers resulted in a successful infusion of morphine (active lever), and the other had no consequence (inactive lever).

### 2.4 Faecal Collection

Fresh faecal samples were collected from awake animals at three different time points throughout this experiment. Prior to MR imaging, animals were removed from their home cage (with adequate bedding and enrichment) and temporarily placed in a clean cage without bedding and sterilized with alcohol only during faeces collection to retrieve spontaneously secreted faeces from each individual animal. Faecal collection occurred at (1) baseline, 7 days after catheter implantation; (2) acute, 24 h after the second day of self‐administration or the third day of experimentation (no intravenous self‐administration on this day); and (3) chronic, 24 h after completion of the 14 days of self‐administration. Faecal samples were collected and immediately stored at −80°C until DNA extraction.

### 2.5 16S rDNA Sequencing

Microbial sequencing was performed as previously described [17, 18]. Microbial DNA was extracted from 50–100 mg faecal material using the Qiagen DNeasy PowerSoil Pro Kit and the automated QIAcube Connect robot (Qiagen, Carlsbad CA) following the manufacturer's instructions. Additional details are shown in the supporting information.

### 2.6 Bioinformatics Analysis

The generated DNA sequences were analysed using the mothur MiSeq SOP pipeline [19]. Microbial diversity (Shannon diversity and evenness), richness (Chao1) and abundance coverage‐based estimator (ACE) were calculated based on Amplicon sequence variants (ASVs) [20, 21, 22]. Microbial communities between morphine and pellets groups were compared and visualized using UniFrac distances and principal coordinate analysis (PCoA) [23]. The molecular variance analysis (AMOVA) was performed to assess the variability among and within different groups [24]. Additional details are shown in the supporting information.

### 2.7 Diffusion MRI

**FIGURE 1:**
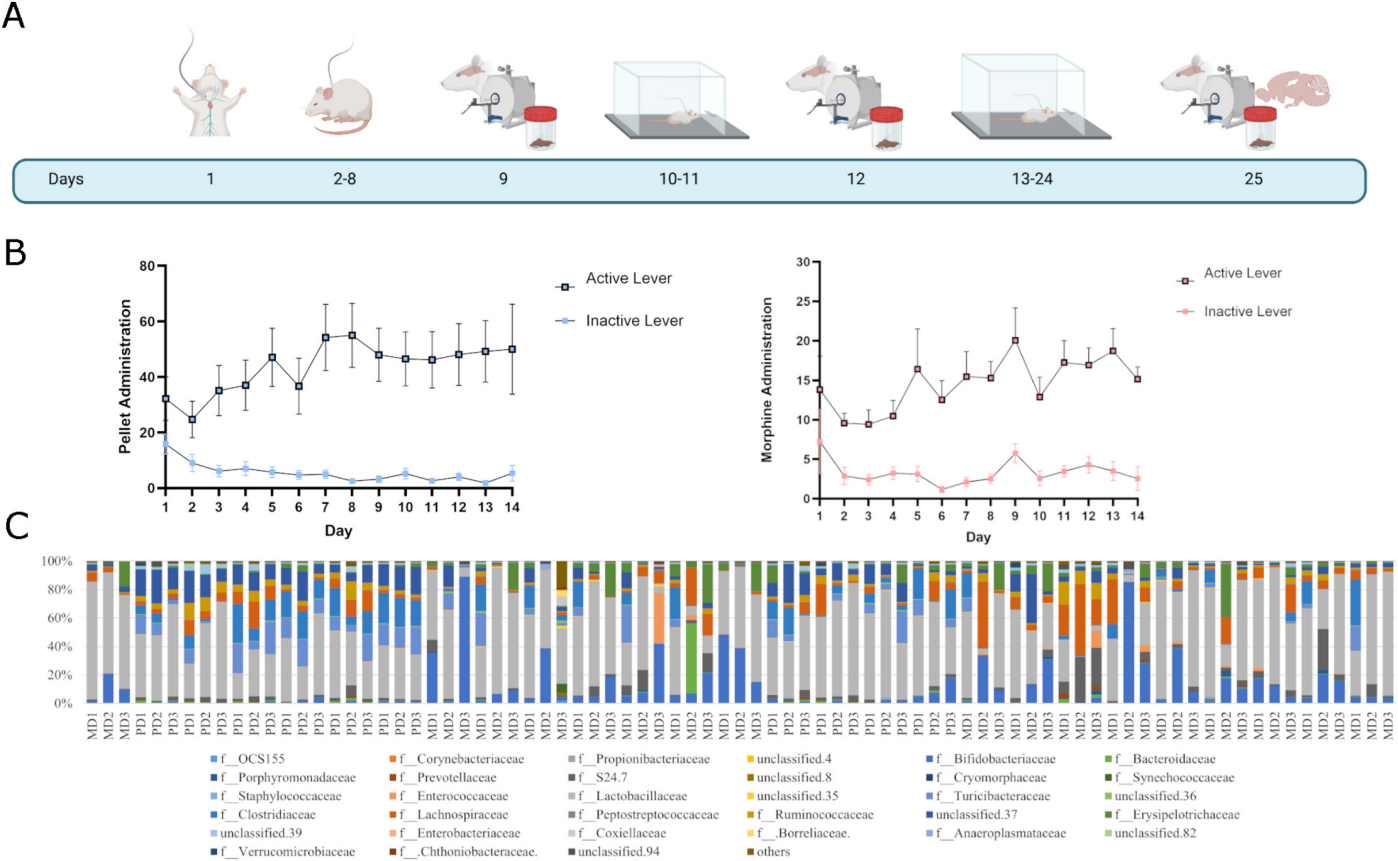
Morphine self‐administration and microbiome composition. (A) Experimental timeline: rats undergo catheter implantation (Day 1) and a recovery period (Days 2–8) before experimental procedures. All rats were probed for baseline gut and brain conditions with faecal collection and MRI (Day 9). Then rats undergo acute self‐administration for 2 days before a second probing session of gut and brain (Day 12), then rats continue to a chronic phase where they undergo SA for an additional 12 days (total of 14 days of self‐administration) before the final and chronic probing sessions that include faeces collection, MRI and brain tissue. (B) Self‐administration: a daily average of infusions or pellet delivery and total number of infusions during the 14 days or pellets delivered. All operant responses showed comparable results for all animals of either sex. (C) Normalized community composition of all animals at all time points at the family level. M = morphine and P = pellets.

Rats were scanned in a cryogen‐free MRI 7T magnet (MRS*DRYMAG7017, MR Solutions, UK). All rats underwent three imaging sessions on the same days as faecal collection (Figure 1A). Diffusion‐weighted images were collected using a two‐shot spin‐echo planar imaging (EPI) sequence with the following parameters: 2 diffusion weighting shells of 18 directions with Jones arrangement [25] at *b* = 500, 60 Jones with *b* = 900 s/mm^2^ and 4 *b* = 0 images. Anatomic scans for image overlay and reference‐to‐atlas registration were collected using a FLASH 3D sequence (spatial resolution = 0.27 × 0.25 × 0.3 mm^3^). Additional details are shown in the supporting information.

### 2.8 DTI Image Processing, Analysis and Correlations

Diffusion data were stripped of the skull and corrected for field inhomogeneities (FSL's topup). The DWI image was registered to the SIGMA rat brain template using ANTs to perform voxel‐based analysis and seed‐based analysis. We generated DTI indices (FSL's dtifit): fractional anisotropy (FA), mean diffusivity (MD), radial diffusivity (RD) and axial diffusivity (ad). The voxel‐based analysis was completed in an in‐house template of FAs, including the baseline data from both groups. Statistical analyses were completed using Python's NumPy and SciPy packages with voxel‐wise *t*‐tests (alpha = 0.05) followed by family‐wise error (FWE) rate correction using the Benjamini–Hochberg false discovery rate into *q*‐values < 0.05. Finally, we registered the SIGMA labels onto the in‐house template and back to the native space of each individual sample to perform seed‐based analysis. Seed‐based analysis was analysed using two‐way repeated‐measures ANOVA and post hoc Tukey multiple test comparisons. Additional details are shown in the supporting information. We estimated correlation between gut and DTI markers using Pearson correlation in R within ggplot.

## 3 Results

### 3.1 Morphine Self‐Administration

To establish volitional morphine use, rats were trained in operant responding to choose an active lever for an infusion of morphine over an inactive lever. Male rats self‐administered13.0 ± 1.8 daily infusions of morphine and females self‐administered 18.4 ± 12.9 daily infusions (Figure 1B). Overall, females self‐administered on average 257.8 ± 181.3 infusions throughout the 14 days, and males self‐administered 182.4 ± 24.6 (*p* = 0.468, Figure 1B). The control group also successfully learned to press levers for sucrose pellets, with daily averages of 46.1 ± 27.9 pellets for males and 45.3 ± 14.4 pellets for females (Figure 1B). Overall, females consumed 645.2 ± 391.2 pellets over the 14 sessions and males 589.2 ± 186.6 (*p* = 0.783, Figure 1B). There was no significant difference in drug consumption between sexes; hence, going forward, we aggregate all results in terms of morphine vs. the control group.

### 3.2 Gut Microbiome

**FIGURE 2:**
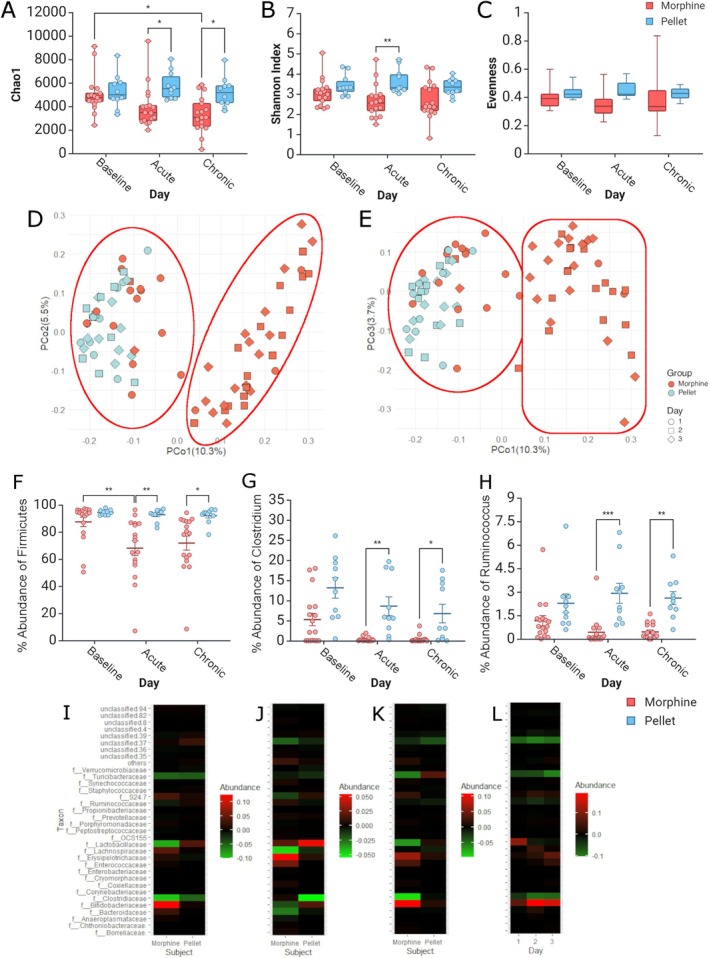
Diversity changes and clustering of microbes due to morphine SA. (A) Chao1, (B) Shannon index and (C) evenness results reflecting relative abundances for each timepoint (i.e., baseline, acute and chronic) and group (i.e., morphine vs sugar pellet). Principal coordinate analysis (PCoA) based on unweighted UniFrac distances plotted as (D) PCo1 vs. PCo2 and (E) PCo1 vs. PCo3. (F) Phylum changes in relative abundance for Firmicutes. (G, H) Genus changes in relative abundances of (G) *Clostridium* and (H) *Ruminococcus 1*, both members of the phylum Firmicutes. (I–L) Differences in relative abundances of taxa at the family level. (I) Acute minus baseline (red acute higher concentration and green baseline higher concentration), (J) Chronic minus baseline (red chronic higher concentration and green baseline higher concentration) and (K) Chronic minus acute (red chronic higher concentration and green acute higher concentration). (L) Comparison of group differences (morphine minus saline, red morphine higher and green pellet higher) for each day. *p < 0.05 for post hoc Tuckey multiple comparisons.

We then sought to determine the effects of morphine self‐administration on gut health via changes in microbial diversity on fecal samples. Bacterial DNA analysis of faecal matter showed microbial diversity changes in rats self‐administering morphine. There were significant differences in alpha diversity measures across days among the pellet and morphine groups using two‐way ANOVA repeated measures. Chao1 showed a progressive reduction in diversity in treatment (*F* = 14.09, *p* = 0.0003). A post hoc test showed a significant difference in abundance between baseline and the chronic stage in the morphine group (*p* = 0.024) but not for the sucrose pellet (Figure 2A). Chao1 also displays differences between groups at the acute stage (*p*=0.048) and chronic stage (*p* = 0.031, Figure 2A). The Shannon index showed a significant effect of treatment (*F* = 16.9, *p* < 0.001) with posthoc significance at the acute stage only (*p*=0.010, Figure 2B). The Evenness index showed no significant changes across days or treatment (Figure 2C). In terms of beta diversity, there were noticeable visual separations in the principal coordinates analysis (PCoA, highlighted areas in Figure 2D,E) across days and whether they administered morphine or sugar pellets with the baseline and of the morphine group aligning with all stages of the sugar pellets between PCo1 and PCo2 (Figure 2D) and between PCo1 and PCo3 (Figure 2E).

Diversity alteration reflected changes at the different taxonomic levels. We found that the phylum *Firmicutes* showed a significant effect of treatment (*F* = 21.9, *p* < 0.0001), with a posthoc difference in percent abundance within the morphine group between baseline and the acute phase (*p* = 0.009) and between groups at the acute phase (*p* = 0.003) and chronic stage (*p* = 0.026, Figure 2F). The phylum Bacteroidetes did not show any differences at any stage or between groups (Figure S1B). The family *Erysipelotrichaceae* showed an effect of treatment (*F* = 6.12, *p* = 0.016) substantial changes in abundance within the morphine group between baseline and chronic stages (post hoc *p* = 0.039, Figure S1D). The families *Ruminococcaceae* (Figure S1E) and *Lactobacillaceae* (Figure S1F) did not show any significant differences in abundance across treatments or days. The differences between taxa at the family level show that the alterations are more prominent for the morphine group than controls (brighter colours of red and green in Figure 2I–K). The between‐group differences at the family level between groups and stages show that differences become more prominent after the baseline when animals self‐administer morphine (Figure 2L).

The phylum and family alteration reflect changes were also observed at the genus level. The genus *Clostridium* showed an effect of treatment (*F* = 36.0, *p* = < 0.0001) significant changes in abundance between groups at the acute stage (post hoc *p* = 0.004) and chronic state (post hoc *p* = 0.049, Figure 2G). The genus *Ruminococcus.1* showed an effect of treatment (*F* = 39.2, *p* < 0.0001) significant difference between groups at the acute stage (post hoc *p* < 0.001) and chronic (post hoc *p* = 0.002, Figure 2H). The genus *Bifidobacterium* showed an effect of treatment (*F* = 15.7, *p* = 0.0001) alterations between groups at the acute stage (*p* = 0.030, Figure S2C). The genus *Turicibacter* showed significant changes in percent abundance between groups at the chronic stage (*p* = 0.001 Figure S2D).

### 3.3 MRI

**FIGURE 3:**
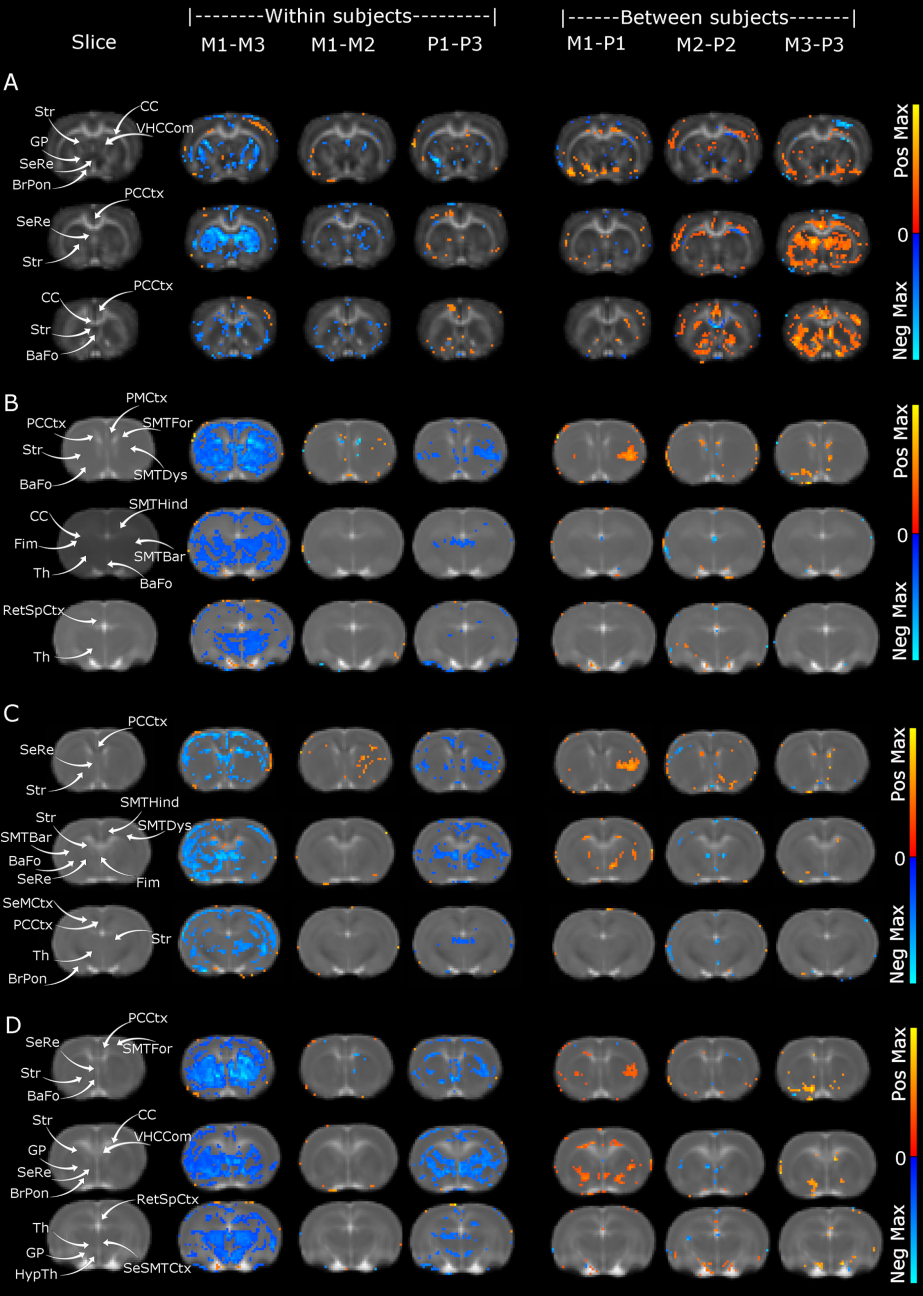
Voxel based differences of DTI maps. (A) The morphine treatment induced chronic change in FA, confirmed by a between groups change in striatum. The acute and pellet state did not induce extensive alterations. (B) MD longitudinal changes were most extensive between chronic and baseline groups in the morphine and pellet treatment comparisons, but morphine induced more widespread changes than pellets. MD differences did not show extensive changes in the between subject's tests. (C) ad longitudinal changes were most extensive between chronic and baseline groups in the morphine and pellet treatment comparisons, but morphine induced more widespread changes than pellets. (D) RD longitudinal changes were most extensive between chronic and baseline groups in the morphine and pellet treatment comparisons, but morphine induced more widespread changes than pellets. RD differences did not show extensive changes in the between subject's tests. PCCtx = primary cingular cortex, SeRe = septal region, Str = Striatum, CC = corpus callosum, BaFo = basal forebrain region, PMCtx = primary motor cortex, SMTFor = somatosensory cortex forelimb, SMTDys = somatosensory cortex dysgranular, SMTBar = somatosensory cortex barrel field, SMTHind = somatosensory cortex hindlimb, Fim = fimbria, Th = thalamus, RetSpCtx = retrosplenial cortex, VHCCom = ventral hippocampal commissure, BrPon = brachium pontis, SeSMTCtx = secondary somatosensory cortex, Th = thalamus, HypTh = hypothalamus, GP = globus pallidum, PVCtx = primary visual cortex, SeMCtx = secondary motor cortex, M1 = Morphine group baseline, M2 = Morphine acute, M3 = Morphine chronic, P1 = pellet group baseline, P2 = pellet group acute, P3 = pellet group chronic. Voxels with intensity are *t* > 2.15, *q* < 0.05, FWE corrected with BH FDR.

Then, we sought to determine the temporal effects of morphine self‐administration on features of brain microstructure using MRI and diffusion tensor imaging (DTI). Here, we describe parallel changes in the brain to those described in the microbiome; we obtained dMRI dataset on the same days faecal samples were acquired. The MRI data were acquired after the faecal collection to minimize acute effects of stress and the scanning in the faecal samples. Voxel‐based analysis of DTI indices showed longitudinal changes within the morphine and pellet self‐administering groups along with between groups comparison at the chronic stage. The FA changes were observed for frontal and striatal regions (Figure 3A). The striatum displays a robust within effect between acute and chronic stage, but not between baseline and acute. Between subject shows a stark contrast in the chronic stage replicating within subjects' findings. Other areas showing differences are the septal region, the primary cingular cortex (PCC) and the corpus callosum (CC). The pellet group did not show large differences from the morphine group, and the early stages of the between‐subjects test also did not show major alterations between groups.

The MD changes resemble alterations observed for FA, with major differences within subjects but not at the between‐subject chronic stage (Figure 3B). The lack of between‐subjects' alterations for the pellet group is further explained as the pellets within subjects' alterations between baseline and chronic are prominent for the similar areas of the morphine group. The MD differences are again prominent for the striatum and PCC for the FA. In addition, we observe the thalamus, basal forebrain region, primary motor cortex (PMC), fimbria, retrosplenial cortex and somatosensory cortices (SMT, dysgranular, hindlimb and barrel field). The main differences in MD are widespread in the comparison within subjects for both groups, with morphine inducing larger effects than sucrose pellets. The within‐subject test for the acute stage did not show changes implying an effect of chronic consumption of morphine or sucrose, with the thalamus exclusively showing differences for morphine.

The ad changes (Figure 3C) are the most prominent but maintain anatomical correspondence with FA and MD. In addition to frontal and striatal regions, we identified alteration in ad for the brain stem (locations where ventral tegmental area, dorsal raphe, pontine nucleus and ventral periaqueductal grey areas). As with the prior test, we again observe major differences in the within‐subject test but less between‐subjects. We observe the striatum and PCC, in addition to the septal region, basal forebrain region, globus pallidum, brachium pontis, thalamus, hypothalamus, SMT's forelimb areas, ventral hippocampal commissure, CC, retrosplenial cortex, secondary SMT and primary visual cortex. The within‐subject test for the acute stage did not show changes implying an effect of chronic consumption of morphine or sucrose, as in the MD test.

The RD (Figure 3D) resembles changes discussed for the MD, albeit less extensive. The within‐subject test for the acute stage did not show changes implying an effect of chronic consumption of morphine or sucrose, and there were no significant changes between‐subjects. Areas displaying alterations in the RD are the striatum, SMTs hindlimb, dysgranular, and barrel field areas, basal forebrain, septal region, brachius pontis, secondary motor cortex and thalamus.

**FIGURE 4:**
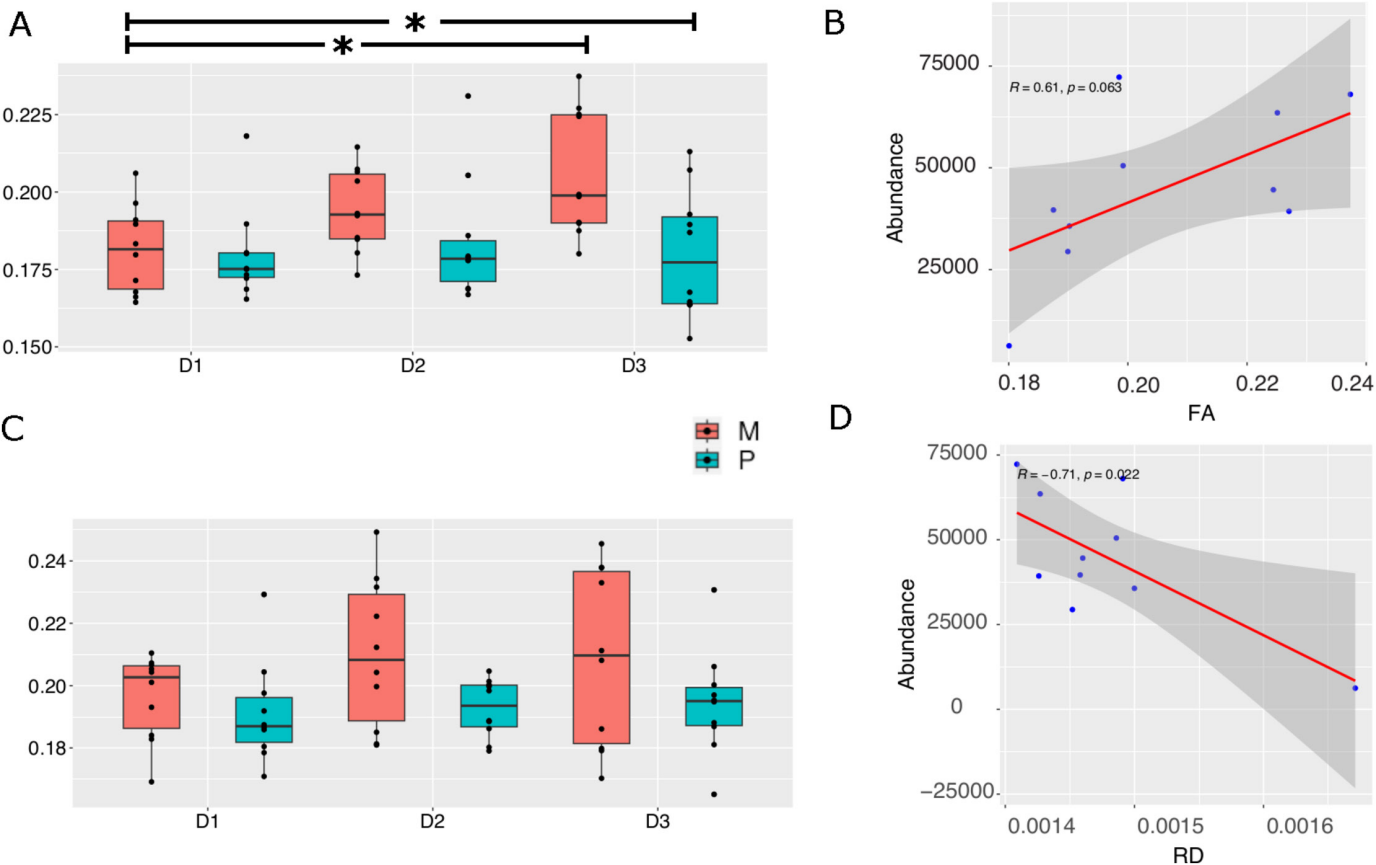
Seed based DT analysis and correlations. (A) Longitudinal changes in striatum FA, and (*F* = 7.66, *p* = 0.007) (B) its correlation between Firmicutes abundance and striatum FA values at the chronic stage of the morphine SA. (C) Longitudinal changes in thalamus FA (*F* = 6.12, *p* = 0.02). (D) Correlation between RD and striatum at the chronic stage of the morphine SA. Stats: two‐way repeated measures ANOVA, *p < 0.05 for posthoc Tuckey tests.

To confirm the results of the voxel‐based analysis, we performed template‐based segmentation of the thalamus and striatum and estimated DTI indices results over the entire region of interest. The prominence of the thalamus and striatum was confirmed by seed‐based analysis using the ROIs from the SIGMA atlas (Figure 4). The FA in the striatum showed an effect of group (*F* = 7.66, *p* = 0.007) and a trend for the interaction between days and group (*F* = 2.42, 0.098). The posthoc analysis showed a statistical difference between morphine at baseline and chronic stage (*p* = 0.03, Figure 4A) and chronic stage between morphine and pellets (*p* = 0.02, Figure 4A). The FA in the thalamus showed an effect of groups (*F* = 6.12, p = 0.02, Figure 4C) without any statistical differences in postdoc test. The MD, ad and RD did not show any statistical differences in the seed‐based analysis.

### 3.4 Correlations

This gut microbiome analysis showed a prominent reduction in the total abundance of Firmicutes in the acute and chronic stages (Figure 2F). Firmicutes reductions imply that there is a reduction in SCFA production, which has been observed in human opioid users, and SCFA has been suggested as a potential therapeutic for opioid use. We then performed a correlation analysis between the Firmicutes positive findings of the gut microbiome and DTI indices at the chronic stage (third MRI) to determine if these concurrent changes show an association at the chronic stage. The correlation analysis between the Firmicutes abundance and striatum RD shows a negative association (*r* = 0.7, *p* = 0.02, Figure 4D), while the striatum FA shows a positive trend (*r* = 0.6, *p* = 0.06, Figure 4B). These associations were not seen at baseline nor at the chronic stage of the pellet group, suggesting that the reduction in Firmicutes abundance and striatum microstructure may be influencing each other.

## 4 Discussion

To the best of our knowledge, this is the first article that concurrently quantifies parallel in vivo and noninvasive changes in the brain and gut microbiome in rodents self‐administering morphine. We present several gut microbiome changes in diversity and taxa, along with longitudinal alterations in brain microstructure due to morphine self‐administration. Our analysis suggests that the striatum is a susceptible area to alterations in the gut due to morphine use. A subset of animals who underwent cellular analysis finds that rats self‐administering morphine show neuroinflammation as a pathological mechanism of bidirectional changes in the gut and brain. Our work further supports the hypothesis of bidirectional communication between the brain and gut as a mechanism of simultaneous changes due to opioid use and hints at a spatiotemporal trajectory to explore in future studies. Our findings show that gut microbiome changes precede changes in brain microstructure assessed with dMRI, suggesting that the gut microbiome may possibly be a putative early marker of GBA dysfunction due to morphine use, over MRI microstructure and that the earliest brain targets associated with gut dysbiosis may be the striatum and thalamus. Several studies have looked at the effects of self‐administration of opioid agonists such as fentanyl [26, 27] and heroin [28]; however, no studies have examined the impact of the microbiome in addiction paradigms for morphine specifically [29]. In the present study, we show a significant decrease in *α* diversity measures among rats that self‐administer morphine, suggesting that the gut‐brain bidirectional communication may have a role in morphine use.

OUD is a critical health crisis in need of novel interventions [30]. The dopaminergic pathway is fundamental to comprehending the neurobiology of opioid use and OUD; however, the GBA has offered a new appreciation of the overarching biological alterations of opioid use and OUD [31]. Opioid agonists also alter *β* diversity, showing distinct features of the microbiome composition following use [32]. Subjects receiving addiction treatment show that opioid agonists (i.e., heroin and prescription opioids) are associated with lower gut microbiome diversity, a finding not observed in those subjects who used agonist + antagonists (i.e., naltrexone), antagonists only and neither agonists nor antagonists (i.e., other substances not targeting opioid receptors) [33]. This work highlights that opioid agonism, and not antagonism or combined administration, impacts gut health, suggesting that the gut microbiome is particularly sensitive to opioid agonism. The opioid agonism is a concern particularly in the treatment of pain with opioids, where opioid antagonists cause gut dysbiosis and constipation. Adjunct treatments that include peripheral opioid antagonists may be candidates to gain nociception with reduced secondary gastrointestinal effects [34]. Gut microbiome depletion via antibiotics enhances fentanyl self‐administration, while repletion of microbial metabolites via short‐chain fatty acid administration reduces it [26, 27]. Our work further supports the finding that opioid agonists alter gut health, leading to a reduction in bacterial diversity.

A mechanism by which opioid agonists alter the microbiome diversity may be through alteration in gut lining affecting permeability. Morphine upregulates toll‐like receptor (TLR, specifically TLR‐2 and TLR‐4) expression levels in small intestinal epithelial cells, sensitizing the small intestinal epithelial cells to TLR stimulation [35], which induces disruption of tight junctions between epithelial cells, increasing gut permeability and bacterial translocation [35]. Morphine chronic administration has been shown to reduce the diversity of Bifidobacteria and Lactobacillaceae and probiotics enhancing these communities improves behaviour [29]. Morphine analgesic tolerance is associated with the abundance of *Ruminococcus 1* [36]. Rats chronically administered morphine display reductions in the genus *Bifidobacterium* during withdrawal, and these microbiome alterations have been suggested to be related to inflammatory changes in the amygdala [37]. The pro‐inflammatory state induced by morphine may be due to an altered Firmicutes‐Bacteroidetes ratio leading to gut barrier breakdown and inflammation [38]. In this work, we find decreases in Firmicutes and increases in Bifidobacteriaceae and Erysipelotrichaceae. At the genus level, we observed reduced *Clostridium*, *Ruminococcus 1* and *Turicibacter* with increased *Bifidobacterium*. A contrast to all other references discussed here is our use of volitional IV self‐administration. In contrast, all references use either a passive administration or oral consumption of water or subcutaneous pellets, which makes our result potentially more relevant for a substance use model.

Observations from MRI correlate with gut composition and behaviour performance in healthy volunteers treated with probiotics in a randomized clinical trial [39]. The prior study proves the potential of gut microbiome diversity enhancement in promoting beneficial neural and behaviour features quantifiable with MRI. The health benefits of adequate gut microbiome composition is not only for healthy subjects, but also schizophrenia subjects, who show reduced microbiome diversity mirror changes in structure and function measured with MRI [40]. Brain microstructure is usually assessed with DTI (i.e., FA, RD, ad and MD indices) and is associated with diet‐dependent changes in gut microbiota populations, such as *Roseburia* and Barnesiellaceae [41]. Gut microbiota from human subjects with ADHD implanted in mice induce anxiety behaviour, and brain microstructure indices of FA and MD in the hippocampus correlate to the abundance of *Eubacterium* [42]. The gut diversity of obese subjects correlates with the FA in the hypothalamus, caudate nucleus, and hippocampus. Also, the abundance of Actinobacteria is associated with DTI indices in the thalamus, hypothalamus, amygdal and cognitive markers of attention and flexibility [43]. These works highlight the reported relationships between DTI and gut microbiome; however, we did not find a reference that highlights studies of morphine and gut microbiome. Our work is the first to identify several changes in the gut microbiome concurrently with changes in DTI indices, such as a prominent change in the striatum and thalamus.

The striatum is a recipient of signalling for the GBA in food intake [44] and Parkinson's disease [45]. GBA alterations are related to dopaminergic alterations in the striatum, like altering transporter expression [45] and altering dopaminergic effluxes [44]. Compulsive alcohol use alters striatal dopaminergic signalling by a reduction of D2 receptor expression in the striatum and a reduction in firmicutes abundance [46]. The alterations in dopaminergic systems may be related to neuroinflammation linking gut dysbiosis to striatal changes due to OUD, implying that complementary gut and brain health markers can be an essential tool for assessing neural health. A possible mechanism might be due to drugs of abuse damaging the gut lining, in turn allowing the transfer of bacterial and pathological compounds to induce neuroinflammation [47, 48, 49] with an emphasis on the striatum. Gut disturbances map neuroinflammation signatures to relevant reward circuitry [50]. Neuroinflammation will cause changes in axonal integrity, and structural connectivity and diffusion imaging studies could provide insights into the potential influence of microglia and neuroinflammatory cascades in neuropsychiatric disorders [51]. Deficits in intestinal permeability, the gut microbiota may induce inflammation and altered behaviour [49]. Cocaine alters the gut‐barrier composition of the tight junction proteins while also impairing epithelial permeability by upregulating proinflammatory cytokines NF‐κB and IL‐1β [52]. Repeated administration of methamphetamine induces proinflammatory cytokine secretion in the medial prefrontal cortex, striatum and hippocampus [53]. In our work, we observe several of these brain regions discussed above but a prominent alteration in striatal microstructure. Although we offer some preliminary relationship between the observed brain changes and gut microbiome changes through neuroinflammation, there are several outstanding gaps in these links. A preliminary analysis (see Supporting Information) shows that microglia may be altered in a subset of the animals used in this study. This observation agrees with previously discussed works that establish that neuroinflammation is an effect of the chronic use of drugs of abuse. We only focused the preliminary immunohistochemistry assay on microglia and astrocyte as their morphology and density may be the most important proxies to interpret in vivo noninvasive and translational metrics from DTI; we recognize the importance to evaluate macrophage, microglia and astrocyte activation markers (CD11b, CD68, CD38, CD80, CD86, among others [54, 55, 56, 57]) that would provide impactful insights for future therapeutics. In addition, such analysis could provide insights between central and peripheral signalling that leads to the inflammatory response. For example, CD45 is an important blood biomarker that could provide insight into the T and B cell immune activation [58]. Also, gaining new insights regarding the immune response will be imperative to determine if MRI is sensitive to specific inflammatory pathways or whether they are general biomarkers of immune response.

In our study, we pooled data of male and female rats together due to lack of self‐administration differences (Figure 1B). Several reports indicate that there are phenotypic differences in self‐administration of drugs in male vs female rats [59, 60, 61]. Potentially, the short and subchronic design may contribute to it, although we did observe a faster active lever presses; overall, the behaviours were similar, which confirms the nuisance of opioid self‐administration where several works report differences while others do not [62]. Potentially stronger opioid agonists such as heroin or oxycodone and more chronic paradigms such as extended access may provide a stronger sex‐dependent effect. Also, several reports highlight sex differences in MRI‐derived markers during development, showing a consistent larger volume of 10% in males [63]. A similar finding is described in terms of white matter maturation using DTI, with male and female subjects showing age‐ and sex‐dependent effects in DTI indices. In our work, pooling our data allows us to consider these contributions to provide insights about morphine‐specific alterations. Finally, the gut microbiome displays sex differences [64]. These alterations in behaviour, brain and gut microbiome are all important risk and sex‐specific considerations for disease and mental health disorders, such as opioid use. In our study, we include data from both sexes to increase the validity of results as sex‐independent outcomes. Future work would disentangle and identify how sex impacts these outcomes and their relationships.

Our work shows several strengths that make it unique, like the reconciliation of in vivo brain features from MRI to longitudinal gut microbiome analysis. Still, we recognize certain limitations of our study. We employ a HARDI (78 diffusion directions) multishell sequence (2 b values), which is superior and the ideal method to discriminate anisotropy features in the brain [25, 65]. We utilized a relatively low diffusion weighting (*b* = 500 and 900), which was chosen to accentuate a higher signal‐to‐noise ratio for all images. Future work can employ higher b values to gain sensitivity to anisotropic microstructural features. Also, the short access (2 h) and relatively short exposure (14 days) of self‐administration afford lower total drug consumption and potentially milder effects than more compulsive elicits methods such as long access or intermittent access and at least 4 weeks of drug self‐administration. The immunohistochemistry validation must be replicated with a larger sample size and with significant and specific markers that would provide meaningful interpretative information and novel therapeutic targets. To maintain safe and healthy catheter IV administration, we used cefazolin as a preventative measure, which has been shown to alter microbiome composition [66]. We feel that the overall consistently higher effect in the morphine group and the extensive literature showing neuroinflammation in several brain areas following administrations of drug abuse make us confident of these results but the specific inflammatory pathways (pro vs. anti‐inflammatory, central vs. peripheral as well).

## 5 Conclusion

With this work, we detected brain and gut changes in vivo and noninvasively in a rodent model of morphine self‐administration. Morphine is notoriously known to alter digestive function and microbiome in humans, as well as in rodent studies using morphine. An understudied aspect of morphine effects in the gut in animal studies is the focus on substance use models, which we attained in this report. In addition, merging MRI with experimental gut techniques, such as 16S rDNA longitudinal sequencing in the same animal, will allow for mapping the trajectory and relationships between GBA and OUD and show that the striatum is a synergistic and vulnerable brain area to the effects of morphine. OUD is one neuropsychiatric disorder that can profoundly gain novel hypotheses and breakthrough insight by leveraging MR and GBA research. It is by joining these two in animal studies of opioid use that we may achieve new transcendental insights about opioid use and OUD that will yield translatable putative biomarkers of drug use that will inform future human studies.

## Author Contributions

Kaylee Brunetti acquired, analysed and interpreted data on this manuscript; Zicong Zhou analysed and interpreted data; Samia Shuchi acquired and analysed data; Raymond Berry analysed and interpreted data; Yan Zhang analysed data; Michael S. Allen acquired data, revised for critical intellectual content and gave final approval; Shaohua Yang acquired and interpreted data, revised for critical intellectual content and gave final approval; Johnny D. Figueroa designed work, interpreted data, drafted work and revised for critical intellectual content, and gave final approval; Luis Colon‐Perez designed the work, acquired, analysed and interpreted data, drafted the work and gave final approval.

## Conflicts of Interest

The authors declare no conflicts of interest.

## Supporting information


**Figure S1.** Phylum and family relative abundances for each timepoint (i.e., baseline = Day 1, acute = Day 2 and chronic = Day 3) and group (i.e., morphine vs sugar pellet). Phylum level changes for (A) Firmicutes and (B) Bacteriodetes. Family level changes for (C) Bifidobacteriaceae, (D) Erysipelotrichaceae, (E) Ruminococacea and (F) Lactobacillaceae. * *p* < 0.05 decrease in Firmicutes and increase in Bifidobacteriaceae and Erysipelotrichaceae.
**Figure S2.** Genus relative abundances for each timepoint (i.e., baseline = Day 1, acute = Day 2 and chronic = Day 3) and group (i.e., morphine vs sugar pellet). (A) *Clostridium*, (B) *Ruminococcus 1*, (C) *Bifidobacterium*, (D) *Turicibacter* and (E) *Allobaculum*. * *p* < 0.05.
**Figure S3.** Immunohistochemistry results. Striatum microglia skeleton analysis. Average number of microglia detected (i.e., Avg # skeletons), number of branches derived from the skeleton (Avg # Branches) and the physical extension of the branches (Overall Avg Branch Length) are shown as the top three plots on the right. Thalamus microglia skeleton analysis is shown in the bottom three plots in the right. *p* < 0.05 Welch’s *t* test.
**Figure S4.** Drug and sugar pellet consumption.

## Data Availability

The data that support the findings of this study are available on request from the corresponding author. The data are not publicly available due to privacy or ethical restrictions.
