# Supplementary material for: Longitudinal and Concurrent Changes in Brain and Gut due to Morphine Self‐Administration
Source: Addict Biol. 2025 Jun 10;30(6):e70059. doi: 10.1111/adb.70059 (PMC12152200; doi:10.1111/adb.70059)
Supplement: Supplementary file 1 — Figure S1. Phylum and family relative abundances for each timepoint (i.e., baseline = Day 1, acute = Day 2 and chronic = Day 3) and group (i.e., morphine vs sugar pellet). Phylum level changes for (A) Firmicutes and (B) Bacteriodetes. Family level changes for (C) Bifidobacteriaceae, (D) Erysipelotrichaceae, (E) Ruminococacea and (F) Lactobacillaceae. * p < 0.05 decrease in Firmicutes and increase in Bifidobacteriaceae and Erysipelotrichaceae. Figure S2. Genus relative abundances for each timepoint (i.e., baseline = Day 1, acute = Day 2 and chronic = Day 3) and group (i.e., morphine vs sugar pellet). (A) Clostridium, (B) Ruminococcus 1, (C) Bifidobacterium, (D) Turicibacter and (E) Allobaculum. * p < 0.05. Figure S3. Immunohistochemistry results. Striatum microglia skeleton analysis. Average number of microglia detected (i.e., Avg # skeletons), number of branches derived from the skeleton (Avg # Branches) and the physical extension of the branches (Overall Avg Branch Length) are shown as the top three plots on the right. Thalamus microglia skeleton analysis is shown in the bottom three plots in the right. p < 0.05 Welch’s t test. Figure S4. Drug and sugar pellet consumption. [file ADB-30-e70059-s001.docx]

**Morphine self-administration alters gut microbiome prior brain changes in the striatum – Supplementary information**

Kaylee Brunetti^1^, Zicong Zhou^1^, Samia Shuchi^1^, Raymond Berry^1^, Sabrina White^2^, Yan Zhang^2^, Michael S. Allen^2^, Shaohua Yang^1^, Johnny D. Figueroa^3^, and Luis Colon-Perez^1*^

^1^ Department of Pharmacology and Neuroscience, University of North Texas Health Science Center, 3500 Camp Bowie Blvd, Fort Worth, TX, 76107, United States of America

^2^ Department of Microbiology, Immunology & Genetics, University of North Texas Health Science Center, 3500 Camp Bowie Blvd, Fort Worth, TX, 76107, United States of America

^3^ Center for Health Disparities and Molecular Medicine and Department of Basic Sciences, Physiology Division, Department of Basic Sciences, Loma Linda University Health School of Medicine, Loma Linda, CA, 92350, United States of America

**Running title: Morphine's effect on gut and brain**

* Corresponding author:

Luis Colon-Perez, PhD

University of North Texas Health Science Center

3500 Camp Bowie Boulevard

Fort Worth, TX 76107

817-735-7679

Email: [luis.colon-perez@unthsc.edu](mailto:luis.colon-perez@unthsc.edu)

Keywords: Morphine, small animal imaging, Diffusion MRI, gut, microbiome, striatum, IV self-administration, addiction.

**Methods**

***Intravenous Self-Administration of Morphine***

Self-administration operant behavior was performed in eight rat behavior chambers (MedAssociates, Fairfax, VT, USA). The chambers were equipped with two retractable levers, and a variable speed syringe pump for infusions. During self-administration sessions, only one of the two levers resulted in a successful infusion of morphine (active lever), and the other had no consequence (inactive lever). The Drug Supply Program of the National Institute on Drug Abuse supplied the morphine used in this study. Morphine hydrochloride was dissolved in 0.9% sterile saline (0.4 mg/kg/infusion). All experiments were performed during the active/dark cycle phase. Before drug self-administration, the animals were pre-trained to lever press for sugar pellets for three days. Once the pre-training was completed, the animals started two-hour sessions of drug self-administration on a fixed ratio schedule of 1 press for drug infusion (morphine, 0.4mg/kg). Rats from either group had to meet a criterion of drug or pellet seeking from day 7 to day 14 but pressed at least seven times for morphine/pellet to be considered for behavior and MRI analysis.

***16S rDNA Sequencing***

Bacterial microbiome sequencing was performed as previously described in.^1,2^ Microbial DNA was extracted from 50~100 mg fecal material using the Qiagen DNeasy PowerSoil Pro Kit and the automated QIAcube Connect robot (Qiagen, Carlsbad CA) following the manufacturer's instructions. We used universal bacterial primers to target the V4 hypervariable region and amplify the 16S rRNA gene ^3,4^. The PCR reaction contained: DNA template (10 ~ 100ng), 0.5 μL of each primer (10 μM), 2.5 μl 10X AccuPrime PCR Buffer II, 2.5 mL BSA (1.6 mg/mL), 1.5 μl Mg (50 mM), 0.1 μL AccuPrime Taq High Fidelity (5U/μL), and PCR grade water to a final volume of 25 μL. PCR amplification was performed in duplicate for each sample and carried out as follows: heated lid 94°C for 2mins, 25 cycles of 94°C for 30s, 52°C for 30s, 68°C for 40s, then 68°C for 5 mins and held at 4°C. The PCR products were confirmed with gel electrophoresis (1.5% agarose), duplicate reactions were combined, and successful products were cleaned using AMPure XP magnetic bead-based purification. We used Illumina Nextera XT Index Kit v2 (Illumina, San Diego, CA), following the manufacturer's instructions, to index the clean PCR products and repurified them with AMPure XP magnetic beads (Beckman Coulter, Chaska, MN). Each 50 μL index PCR reaction contained 5 μL 10X AccuPrime PCR Buffer II, 5 μL Nextera XT indexing primers 1, 5 μL Nextera XT indexing primers 2, 0.2 μL AccuPrime Taq High Fidelity (5U/μL), 5 μL purified DNA, and PCR grade water. The PCR conditions were as follows: heated lid 94°C for 3mins, 8 cycles of 94°C for 30s, 55°C for 30s, 68°C for 30s, then 68°C for 5 mins and held at 4°C. We used the Qubit dsDNA HS Assay Kit (Invitrogen, Carlsbad, CA) to quantify PCR products and pool them in equimolar amounts. The pooled sample was then denatured, diluted, loaded, and sequenced using a Miseq Reagent V2 (500 cycles) kit following the manufacturer's instructions.

**Bioinformatics Analysis**

The generated DNA sequences were analyzed using the mothur MiSeq SOP pipeline^5^. We assembled paired-end sequences and removed short (< 100bp) and low-quality sequences (homopolymers > 8) from the dataset. We used the SILVA database to align sequences. The unaligned sequences and gaps were removed. Redundant sequences were reduced using the unique.seqs command, a precluster (diffs=2) algorithm, and chimeras were removed after identification using UCHIME^6^. We used the Ribosomal Database Project (RDP) classifier with a minimum of 80% confidence to classify taxa ^7^ and the up-to-date curated EzBiocloud database as a reference. We removed sequences classified as mitochondria, chloroplast, archaea, and eukaryote from the dataset.

Microbial diversity (Shannon diversity and evenness), richness (Chao1), and abundance coverage-based estimator (ACE) were calculated based on Amplicon Sequence Variants (ASVs) each representing a unique sequence within the dataset ^8–10^. Microbial communities between morphine and pellets groups were compared and visualized using UniFrac distances and principal coordinate analysis (PCoA)^11^. The Molecular Variance analysis (AMOVA) was performed to assess the variability among and within different groups^12^. Diversity estimators, UniFrac, Principal Coordinate Analysis (PCoA).

***Diffusion Magnetic Resonance Imaging***

Rats were scanned in a cryogen-free MRI 7T magnet (MRS*DRYMAG7017, MR Solutions, UK) All rats underwent three imaging sessions on the same days as fecal collection (Fig 1A). The animals were anesthetized with isoflurane (5% induction, ~1.5% maintenance) and then transferred to the scanner, where temperature and breathing rate were monitored throughout the scan (SA Instruments). A warm air-heated bed maintained the rat's body temperature at 37–38°C. Diffusion-weighted images were collected using a two-shot spin-echo echo planar imaging (EPI) sequence with the following parameters: 2 diffusion weighting shells of 18 directions with Jones arrangement^13^ at b = 500, and 60 Jones with b = 900 s/mm^2^, and 4 b=0 images, echo time (TE) = 25 ms; repetition time (TR) = 5.0 s; 32 × 32 mm in plane; 16 slices with 1.3 mm thickness per slice; data matrix = 100 × 94; phase encode direction IS. We also obtain a single b = 0 blip-down image with phase encoding in the SI direction. Anatomic scans for image overlay and reference-to-atlas registration were collected using a FLASH 3D sequence (TI = 2200 ms; TE = 4.9 ms; TR = 25 ms; number of averages = 3; data matrix = 134 x 128 x 100 and spatial resolution = 0.27 x 0.25 x 0.3 mm^3^).

***DTI image processing and analysis***

Brain masks were generated using high-resolution anatomic scans, and RBM was manually edited to maintain only brain tissue using ITK-SNAP. The cropped brain structural image was aligned with the B0 image of the DWI scan to obtain a brain mask in DWI space using the FMRIB Software Library linear registration program FLIRT. Diffusion data was stripped of the skull and corrected for field inhomogeneities (FSL's topup). The DWI image was registered to the SIGMA rat brain template using ANTs (AntsQuickSyn) to obtain segmentation and perform voxel-based analysis and seed-based analysis. We generated DTI indices (FSL's dtifit): fractional anisotropy (FA), mean diffusivity (MD), radial diffusivity (RD), and axial diffusivity (AD) in native space. The voxel-based analysis was completed in an in-house template of FAs including the baseline data from both groups, using ANTs function antsMultivariateTemplateConstruction2.sh. Then, we proceeded to register all FAs of each sample in both groups and in all days: baseline, acute, and chronic, onto the in-house template-FA, using ANTs function antsRegistrationSyn.sh. Then, we applied the in-house template registration onto MD, AD and RD, so that all DTI maps are warped in the in-house template image space using ANTs function antsApplyTranform.sh. Statistical analysis were completed using Python's Numpy and Scipy packages with voxel-wise t-tests (alpha = 0.05) followed by Family-Wise Error (FWE) rate correction using the Benjamini-Hochberg False Discovery Rate into q-values < 0.05. Finally, we registered the SIGMA labels onto the in-house template and back to the native space of each individual samples to perform seed-based analysis. Seed-based analysis was analyzed using 2-way repeated-measures ANOVA, and posthoc Tuckey testing.

***Tissue Collection and Immunohistochemistry***

The animals were anesthetized with isoflurane and decapitated. Brains were rapidly removed, fixed in 4% formalin, and further processed to be embedded in paraffin. The immunofluorescence protocol used in this study was adapted from Liu et al^14^. Brain sections were then stained with primary antibodies for GFAP (Santa Cruz, 1:200), Iba1 (Cell Signaling, 1:200), and DAPI (Cell Signaling, 1:200) overnight at 4 °C, followed by species-specific secondary antibodies. Microscopic images were acquired with a Keyence BZ microscope and analyzed with FIJI^15^.

***Immunofluorescence analysis***

In our study, we used the FIJI AnalyzeSkeleton plugin to calculate the area covered, branch size, and branch length to characterize cell morphology following morphine IVSA. Results were analyzed with a Welch’s t test^16^.

**Figures**


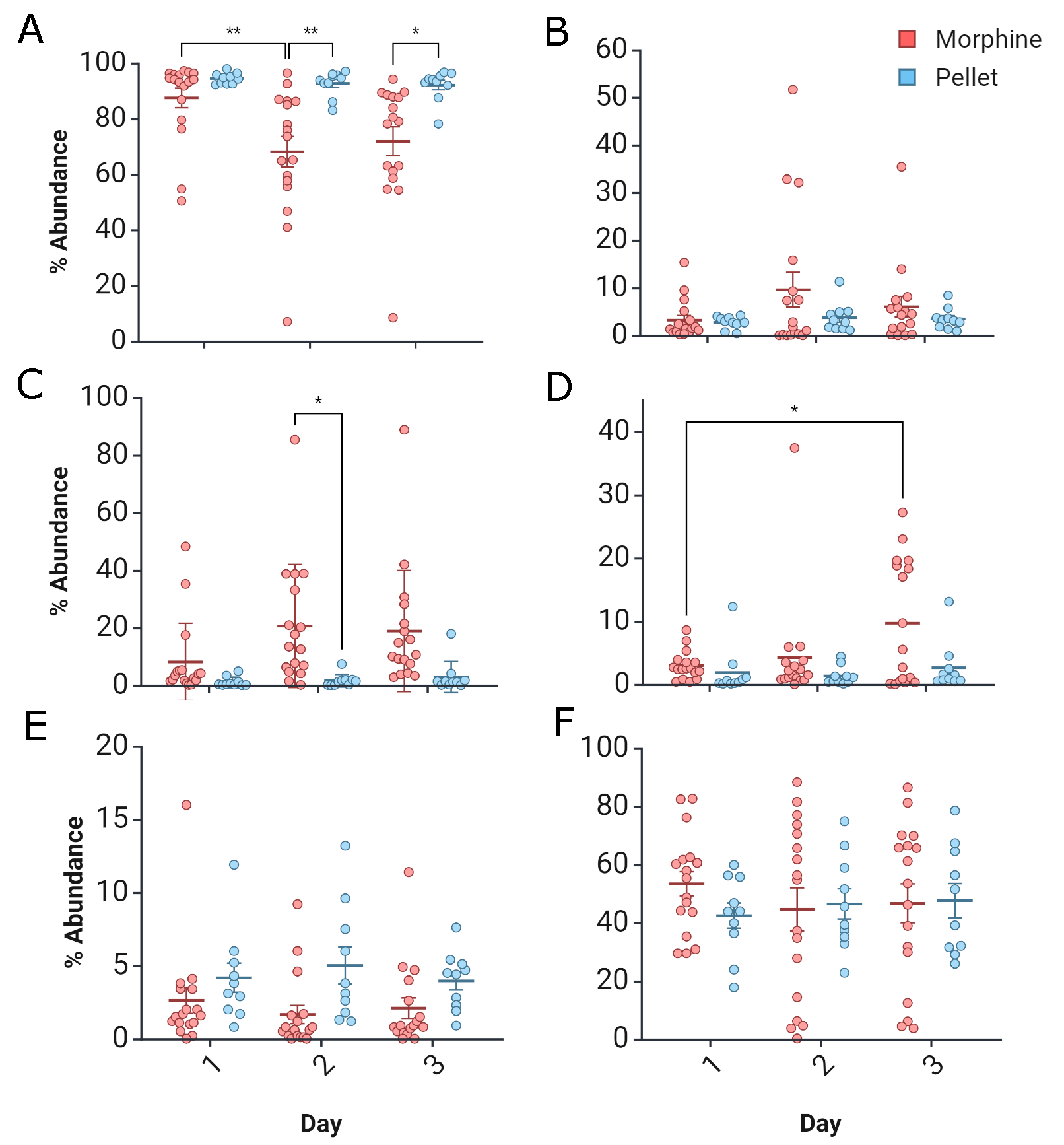


Figure 1. Phylum and family relative abundances for each timepoint (i.e., baseline = Day 1, acute = Day 2, and chronic = Day 3) and group (i.e., morphine vs sugar pellet). Phylum level changes for A) Firmicutes and B) Bacteriodetes. Family level changes for C) Bifidobacteriaceae, D) Erysipelotrichaceae, E) Ruminococacea, and F) Lactobacillaceae. * p < 0.05 decrease in Firmicutes and increase in Bifidobacteriaceae, and Erysipelotrichaceae.


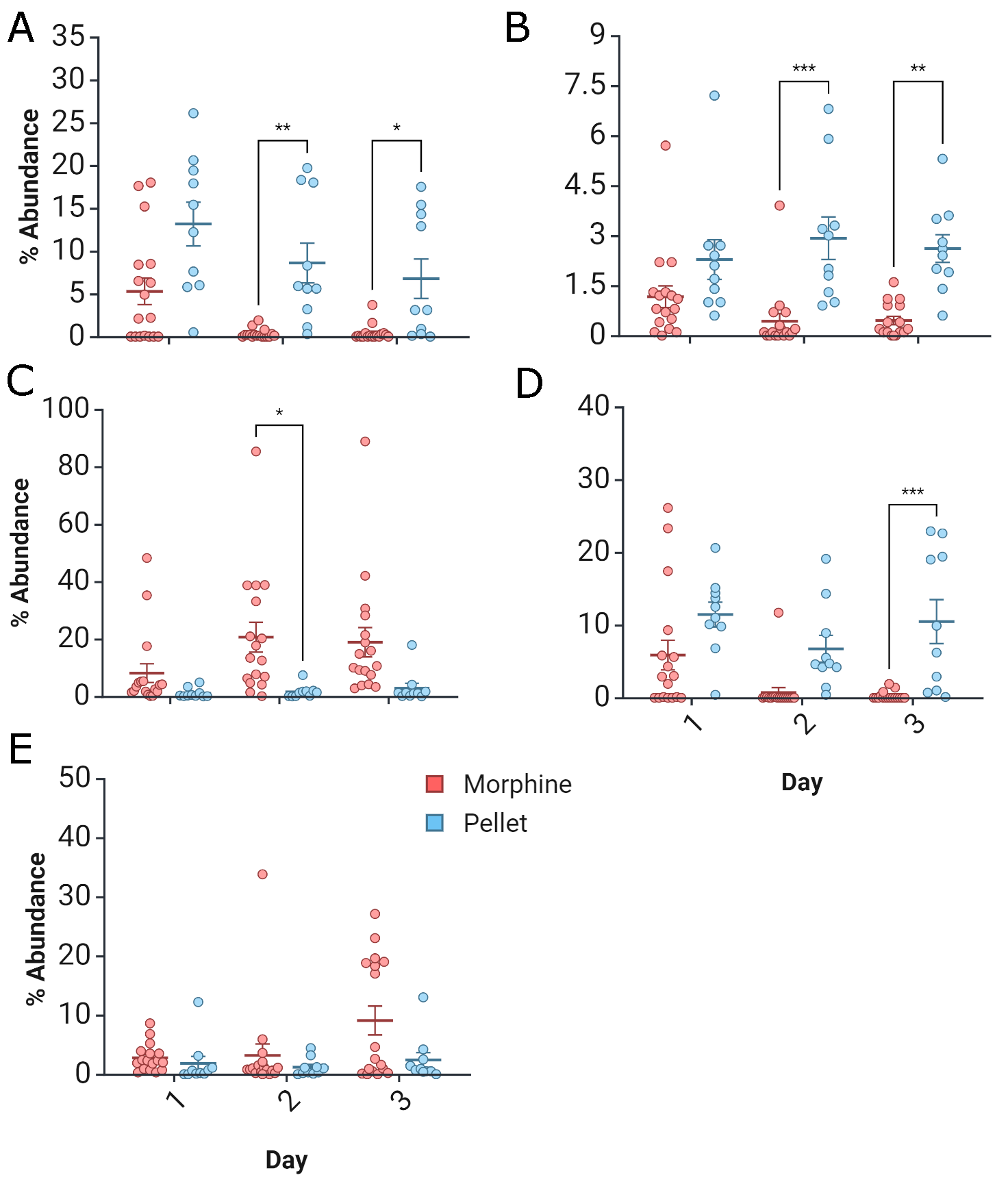


Figure 2. Genus relative abundances for each timepoint (i.e., baseline = Day 1, acute = Day 2, and chronic = Day 3) and group (i.e., morphine vs sugar pellet). A) *Clostridium*, B) *Ruminococcus* *1*, C) *Bifidobacterium*, D) *Turicibacter*, and E) *Allobaculum*. * p < 0.05


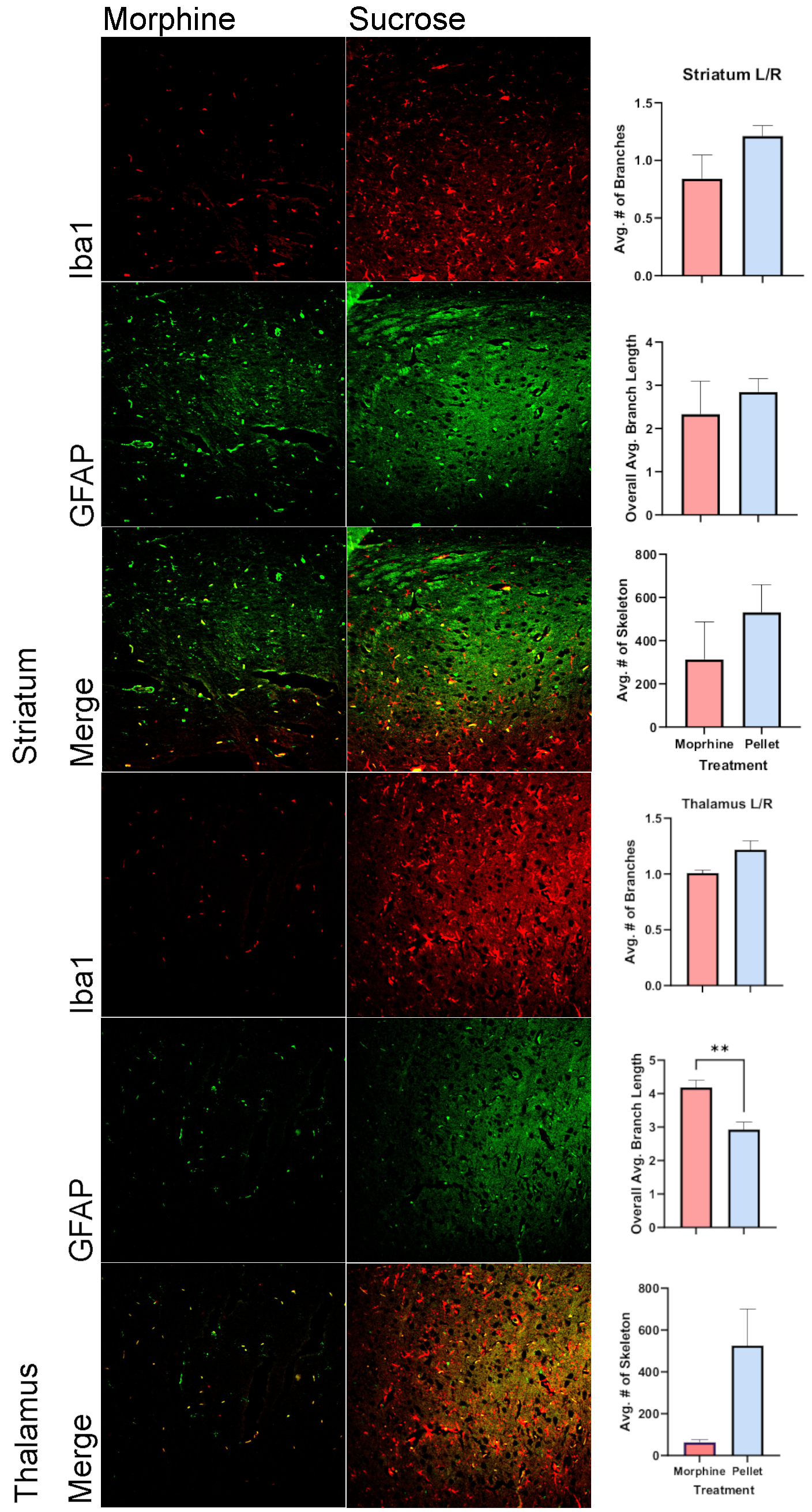


Figure 3. Immunohistochemistry results. Striatum microglia skeleton analysis. Average number of microglia detected (i.e., Avg # skeletons), Number of branches derived from the skeleton (Avg # Branches), and the physical extension of the branches (Overall Avg Branch Length) are shown as the top three plots on the right. Thalamus microglia skeleton analysis is shown in the bottom three plots in the right. p < 0.05 Welch’s t test

***Immunohistochemistry Exploratory Results***

We performed an exploratory small sample exploration to determine whether MRI derived brain changes could be related to a neuroinflammatory response due to morphine use. To determine potential insights on the source of DTI changes, we performed immunohistochemistry of markers of neuroinflammation in the most prominent regions: thalamus, and striatum (Fig. 3). Neuroinflammation has extensively been observed using DTI in humans and rodents, and neuroinflammation may be an outcome of the altered GBA communication leading to pathology. Markers of microglia and astrocytes were done in the thalamus and striatum, displaying a possible effect in markers associated with microglia suggesting that the observation that drug of abuse, including morphine cause neuroinflammation, and in this paper such neuroinflammatory response in rats self-administering morphine may be identified as DTI alterations. The morphine self-administration group displayed reduced area and branching with increased branch lengths (Fig 5G, t =2.47, p = 0.056) (Fig 5I, t 2.5 p = 0.046), Fig 5H, t = 3.9, p = 0.0052). No differences in area were seen for cell nucleus markers or astrocytes.


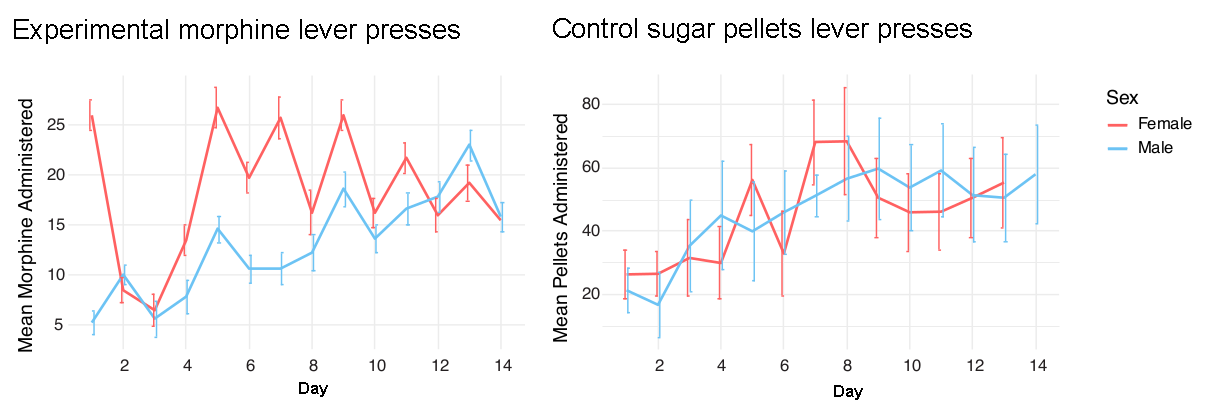


Figure 4. Drug and sugar pellet consumption.

**References:**

1. Thyagarajan, S. *et al.* Comparative analysis of racial differences in breast tumor microbiome. *Scientific Reports 2020 10:1* **10**, 1–13 (2020).

2. Mancilla, V. J. *et al.* A Synthetic Formula Amino Acid Diet Leads to Microbiome Dysbiosis, Reduced Colon Length, Inflammation, and Altered Locomotor Activity in C57BL/6J Mice. *Microorganisms* **11**, 2694 (2023).

3. Apprill, A., Mcnally, S., Parsons, R. & Weber, L. Minor revision to V4 region SSU rRNA 806R gene primer greatly increases detection of SAR11 bacterioplankton. *Aquatic Microbial Ecology* **75**, 129–137 (2015).

4. Parada, A. E., Needham, D. M. & Fuhrman, J. A. Every base matters: assessing small subunit rRNA primers for marine microbiomes with mock communities, time series and global field samples. *Environ Microbiol* **18**, 1403–1414 (2016).

5. Schloss, P. D. *et al.* Introducing mothur: open-source, platform-independent, community-supported software for describing and comparing microbial communities. *Appl Environ Microbiol* **75**, 7537–7541 (2009).

6. Edgar, R. C., Haas, B. J., Clemente, J. C., Quince, C. & Knight, R. UCHIME improves sensitivity and speed of chimera detection. *Bioinformatics* **27**, 2194–2200 (2011).

7. Wang, Q., Garrity, G. M., Tiedje, J. M. & Cole, J. R. Naive Bayesian classifier for rapid assignment of rRNA sequences into the new bacterial taxonomy. *Appl Environ Microbiol* **73**, 5261–5267 (2007).

8. Shannon, C. E. & Weaver, W. *The Mathematical Theory of Communication.* *The mathematical theory of communication.* (University of Illinois Press, Champaign,  IL,  US, 1949).

9. Chazdon, R. L., Colwell, R. K., Denslow, J. S. & Guariguata, M. R. Statistical methods for estimating species richness of woody regeneration in primary and secondary rain forests of Northeastern Costa Rica. Preprint at https://hdl.handle.net/10568/17965 (1998).

10. Chao, A. Nonparametric Estimation of the Number of Classes in a Population. *Scandinavian Journal of Statistics* **11**, 265–270 (1984).

11. Lozupone, C. & Knight, R. UniFrac: a new phylogenetic method for comparing microbial communities. *Appl Environ Microbiol* **71**, 8228–8235 (2005).

12. Anderson, M. J. A new method for non-parametric multivariate analysis of variance. *Austral Ecol* **26**, 32–46 (2001).

13. Jones, D. K., Horsfield, M. A. & Simmons, A. Optimal strategies for measuring diffusion in anisotropic systems by magnetic resonance imaging. *Magn Reson Med* **42**, 515–525 (1999).

14. Liu, R. *et al.* Experimental Ischemic Stroke Induces Secondary Bihemispheric White Matter Degeneration and Long-Term Cognitive Impairment. *Transl Stroke Res* (2024) doi:10.1007/S12975-024-01241-0.

15. Schindelin, J. *et al.* Fiji - an Open Source platform for biological image analysis. *Nat Methods* **9**, 676–682 (2012).

16. Gorlewicz, A. *et al.* Colocalization Colormap -an ImageJ Plugin for the Quantification and Visualization of Colocalized Signals. *Neuroinformatics* **18**, 661–664 (2020).
